# Effects of Beryllium Addition on Microstructure, Mechanical and Corrosion Performance of Al-Mg-Li Alloys

**DOI:** 10.3390/ma16186308

**Published:** 2023-09-20

**Authors:** Yang Huang, Weiwei Li, Mingdong Wu, Daihong Xiao, Lanping Huang, Wensheng Liu

**Affiliations:** National Key Laboratory of Science and Technology on High-Strength Structural Materials, Central South University, Changsha 410083, China; 203112141@csu.edu.cn (Y.H.);

**Keywords:** Al-Mg-Li alloy, beryllium content, microstructure, mechanical properties, corrosion

## Abstract

The Al-Mg-Li alloy is ideal for ultra-lightweight aircraft components, and its further performance improvement is of great interest in the aerospace industry. In this study, the effects of various beryllium (Be) additions (Be-free, 0.1, 0.25, 0.50 wt.%) on the microstructure, mechanical, and corrosion performance of the Al-Mg-Li alloys were systematically investigated. The optimal tensile property was obtained in the alloy which added 0.1 wt.% Be with an ultimate tensile strength (UTS), yield strength (YS), and elongation (El) of 530 MPa, 370 MPa, and 9.2%, respectively. Trace Be addition promotes the grain refinement of the as-cast alloy ingot and contributes positive effects to the recrystallization, bringing improvement of the tensile property. Meanwhile, the best anti-corrosion behavior is also presented at 0.1 wt.% Be is added, due to its potential to reduce the width of precipitates free zone (PFZ). As the Be content increases to an excessive level, the comprehensive performance decreases. Therefore, it is strongly recommended that adding trace Be elements into Al-Mg-Li alloys has a positive effect on the comprehensive service performance.

## 1. Introduction

Adding small amounts of lithium (Li) into aluminum (Al) alloys grants a state-of-the-art combination of comprehensive properties (e.g., high specific strength, outstanding weldability, and good anti-corrosion performance) and weight reduction, which has made Al-Li alloy a desirable structural material in the aerospace industry [1,2,3,4,5,6]. Similar to Al-Cu-Li alloys, Al-Mg-Li alloys are one of the greatest Al alloy series interested in the aero-industrial application, particularly in the development of ultra-lightweight spacecraft parts that demand further weight decrease, because of their extremely low density [7,8,9].

Currently, micro-alloying is the main strategy to boost the processing properties and service performance of Al alloys [10,11,12,13,14]. Therefore, finding an appropriate micro-alloying element to elevate the overall performance of Al-Mg-Li alloys is essential. The element beryllium (Be) is widely used as an additive to raise the performance of casting Al alloys [15,16]. Adding a small amount of Be to the casting of high-quality Al alloys used in the aerospace industry enables a thin film of Be oxides produced on the interface between the melt and air, which effectively reduces molten slag and contributes to the degassing effect, improves the purity of the melt and increases its fluidity, resulting in ingots with high purity and good surface finish [17]. Mortaza et al. investigated the influence of adding trace amounts of Be into the Al-Mg_2_Si composites on the mechanical properties and microstructure and experimental results showed that the number density and average size of the primary Mg_2_Si phases reduced with the elevating of Be addition, improving the tensile strength as well as ductility [18]. Peng et al. also added Be for modifying Mg_2_Si, eutectics, and α-Al matrix in the 15% Mg_2_Si/Al-8Si composites [19].

Moreover, Al-Mg-Li alloys are aging strengthening materials, and their main precipitates are Al_3_Li (δ′), and Al_2_MgLi. Due to the Al_2_MgLi precipitate being located at the grain boundary, it exerts a few effects in strengthening contribution [20,21,22,23]. Therefore, the prominent strengthening precipitate within the Al-Mg-Li alloys is δ′. Though a few previous studies were carried out on the effect of adding Be into Al alloys [18,24], there are lack of elaborate studies focusing on the precipitation behavior of Al-Mg-Li alloys with distinct additions of Be element. Thus, there is still insufficient investigation into how the Be element influences the comprehensive performance of Al-Mg-Li alloy, which requires more research efforts.

In this work, there are four alloys with various Be additions that were selected as the experimental materials. Their mechanical properties and anti-corrosion performance were obtained. The precipitation behavior of each alloy was inspected by transmission electron microscopy (TEM). Electron backscattering diffraction (EBSD) was used to characterize the grain structure and recrystallization behavior of Al-Mg-Li alloys with various Be additions. The purpose of this work is to combine the following influences of various Be additions on the grain structure, recrystallization behavior, and precipitation behavior to guide the subsequent development of new Al-Mg-Li alloys.

## 2. Experimental Procedures

The Al-Mg-Li ingots used in this work were prepared by casting, including four alloys with different Be content. A inductively coupled plasma emission spectrometry (ICP-AES) was used to check their chemical composition. As shown in Table 1, these four alloys are named alloy 1 (Be-free), alloy 2 (0.10 wt.% Be), alloy 3 (0.25 wt.% Be) and alloy 4 (0.50 wt.% Be).

**Figure 1:**
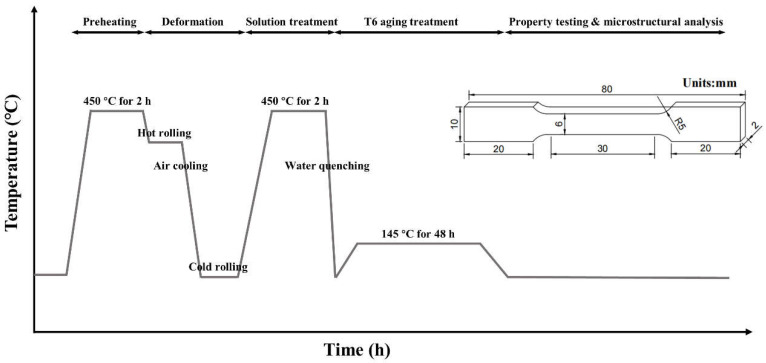
Illustration of experiment route and the dimensions of a tensile specimen.

Initially, these as-cast ingots were enduring a homogenization process for 2 h at 480 °C. After being preheated at 450 °C for 2 h, the homogenized ingots were hot-rolled to sheets with a speed of 5 cm/s and the reduction ratio is about 75%. Their thickness was decreased from 10 to 2 mm during the cold-rolling process. Then, these sheets were put into a furnace for solid solution treatment at 450 °C for 2 h, and water quenched at ambient temperature. Subsequently, the quenched specimens were subjected to a T6 artificial aging treatment of 145 °C for 48 h. The whole experiment process is presented in Figure 1.

Based on the standard GB/T 228.1-2021 [25], the tensile test samples with a dimension of 6 × 2 mm were taken from the above sheet along its rolling direction (RD), where the detailed scales are presented in Figure 1. The tensile test was performed using the Instron 3369 testing machine (Instron, Norwood, MA, USA) at ambient temperature with a strain rate of 0.001 s^−1^. These samples were tested three times for each alloy.

According to GB/T 7998-2005 [26] standards and GB/T 40338-2021 [27], the intergranular corrosion (IGC) tests and exfoliation corrosion tests were conducted to evaluate the anti-corrosion performance. The corrosion-tested surface is the rolling plane for both tests. Prior to the IGC test, the rolling plane of the sample was polished and then immersed in a mixture of 30 g NaCl per liter and 10 mL HCl per liter at ~35 °C for 24 h. As for the preparation of the exfoliation corrosion medium, 53.5 g of NH_4_Cl, 1.84 g of (NH_4_)_2_C_4_H_4_O_6,_ and 20 g of NH_4_NO_3_ were dissolved in distilled water, then 10 mL of H_2_O_2_ was added, and finally this solution was diluted to 1 L with distilled water. The exfoliation corrosion samples were soaked in the above mixture for 24 h. According to GB/T 7998-2005, the corrosion depth into the surface was inspected using optical microscopy (OM).

The rolling plane is the microstructure observation plane for all characterization methods. For EBSD analysis, the samples were initially ground with sandpaper to a smooth surface and polished with Al_2_O_3_ polishing powder. Then, the samples were electrolytically polished with 10% HClO_4_ and a 90% C_2_H_5_OH mixture at −30 °C with an operating voltage of 25 V for 30 s. The CHANNEL-5 and Aztec-crystal software (https://nano.oxinst.com/azteccrystal) were used to carry out data processing. For TEM inspection, the specimens were initially milled to a 70~90 μm foil, and then twin-jet electrolytic thinning was preformed using a mixture containing 25% HNO_3_ and 75% CH_3_OH at −30 °C. The characteristics of precipitates (e.g., fraction, size as well and distribution) were inspected by JEM-2100F, JEOL, Tokyo, Japan.

## 3. Results

### 3.1. Tensile Properties

**Figure 2:**
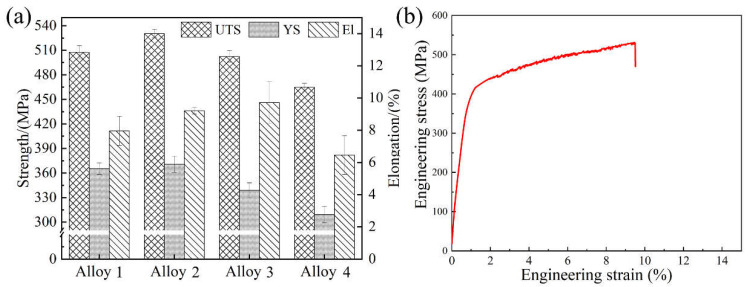
(**a**) tensile properties of alloys with different Be addition; (**b**) engineering stress-strain curve of alloy 2.

Figure 2 exhibits the histograms of the tensile properties of alloys 1 to 4 and the stress-strain curve of alloy 2 under the T6 aging condition of 145 °C/48 h. Detailed data on the tensile properties of each alloy are exhibited in Table 2. It is obvious that alloy 2 has the best overall room temperature tensile properties. The tensile properties of the alloy increased with a minor addition of Be, while it decreased with the excessive addition.

It can be seen that alloy 2 contains 0.1 wt.% Be and exhibits the best performance at 145 °C/48 h aging with UTS, YS, and El of 530 MPa, 370 MPa, and 9.2%, respectively, and the calculated specific strength is 214 MPa·cm^3^/g. In contrast, the Be-free alloy 1 shows UTS, YS, and El of 495 MPa, 331 MPa, and 8.3% after 145 °C/48 h of aging. When the Be content increased to 0.25 wt.%, the UTS and YS of alloy 3 at 145 °C/48 h of aging decreased to 502 MPa and 338 MPa, respectively. Its specific strength is 203 MPa·cm^3^/g, which is significantly lower than that of alloy 2, while El increases to 9.7%. When the Be content continued to increase to 0.50 wt.%, the UTS, YS, and El of alloy 4 at 145 °C/48 h of aging had continued to reduce to 464 MPa, 309 MPa, and 6.5%, respectively, and the specific strength was further reduced to 187 MPa·cm^3^/g.

For short, the addition of 0.1–0.25 wt.% Be shows a positive effect on the room-temperature tensile properties of the Al-Mg-Li alloy. The addition of 0.1 wt.% Be notably improves the strength and elongation, but the excessive addition of Be content causes a remarkable degradation of tensile properties.

### 3.2. Corrosion Behaviors

**Figure 3:**
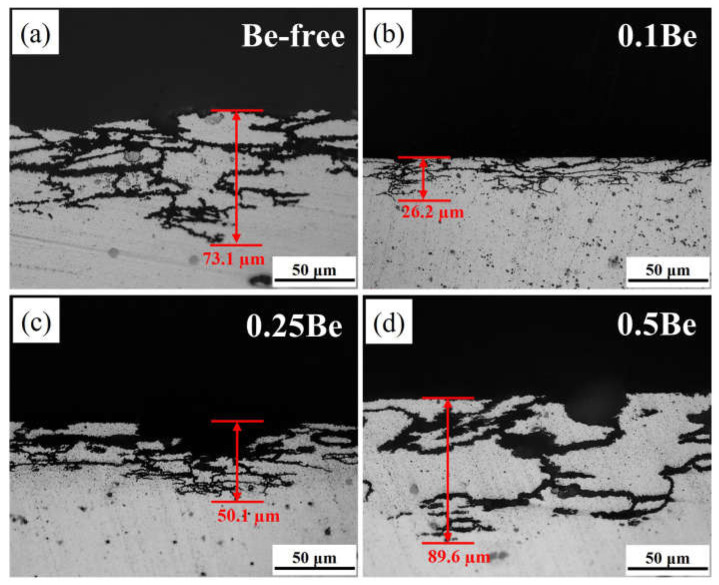
OM graphs of the alloys with various Be additions after the IGC test: (**a**) alloy 1; (**b**) alloy 2; (**c**) alloy 3; (**d**) alloy 4.

Figure 3 presents the OM graphs of the cross-section of alloys 1–4 in 145 °C/48 h of aging condition and after 24 h of immersion in IGC solution. It can be seen that the corrosion mode of all alloy samples is uniform IGC, corrosion extends inside the alloy along grain boundaries. The alloy 4 experienced severe IGC, in which its corrosion surface produced partial detachment where there is corrosion along the large angle grain boundary deep inside the alloy, and the corrosion band is wide with a maximum corrosion depth of 89.6 μm. The corrosion of alloy 1 is also aggressive. The grain of the corrosion surface detached and the corrosion bandwidth is slightly less than alloy 4 with a maximum corrosion depth of about 73.1 μm, which is slightly shallower than alloy 4. Alloy 2 presents the best performance to IGC resistance. But there is no obvious grain detachment on its corrosion surface the corrosion band is thin, and the corrosion depth is the shallowest, with the maximum corrosion depth of only 26.2 μm, which is significantly lower than that of alloy 4 and alloy 1. The IGC resistance of alloy 3 is between alloy 1 and alloy 2, with obvious traces of pitting on the corrosion surface and thin corrosion bands along the grain boundaries in the interior, with a maximum corrosion depth of about 50.1 μm.

**Figure 4:**
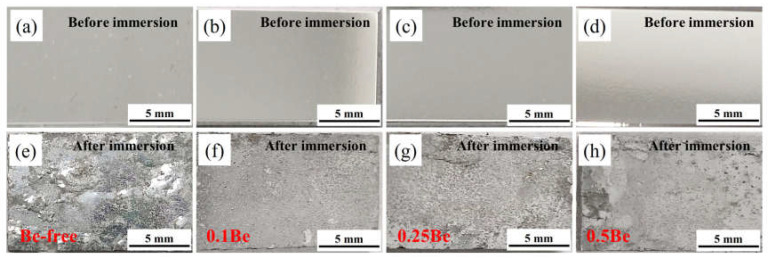
Digital images of the alloys with different Be additions before and after the Exfoliation corrosion test: (**a**,**e**) alloy 1; (**b**,**f**) alloy 2; (**c**,**g**); alloy 3; (**d**,**h**) alloy 4.

The macroscopic pictures of exfoliation corrosion of alloys with different Be content after aging treatment at 145 °C/48 h is shown in Figure 4. The corroded surface of Alloy 1 shows severe pitting, along with skin bursting, blistering, and surface cracking, indicating poor corrosion resistance. The corrosion surface of alloy 2 exhibits minor pitting, no skin bursting or cracking, and shows good anti-corrosion performance. With the excessive addition of Be content, the anti-exfoliation corrosion performance deteriorates. The exfoliation corrosion of alloy 3 shows more serious pitting, while the corrosion surface edge exhibits a blistering and spalling phenomena. On the corroded surface of Alloy 4, the presence of pits, together with the large area of blister scars, cracking, and spalling of the alloy edges, indicate further deterioration in corrosion performance.

In a word, the trace addition of 0.1 wt.% Be can significantly improve the anti-exfoliation corrosion performance of Al-Mg-Li alloys, but if the Be addition in the alloy is too excessive, the corrosion resistance is notably reduced.

### 3.3. Microstructures

#### 3.3.1. As-Cast Grain Structures

**Figure 5:**
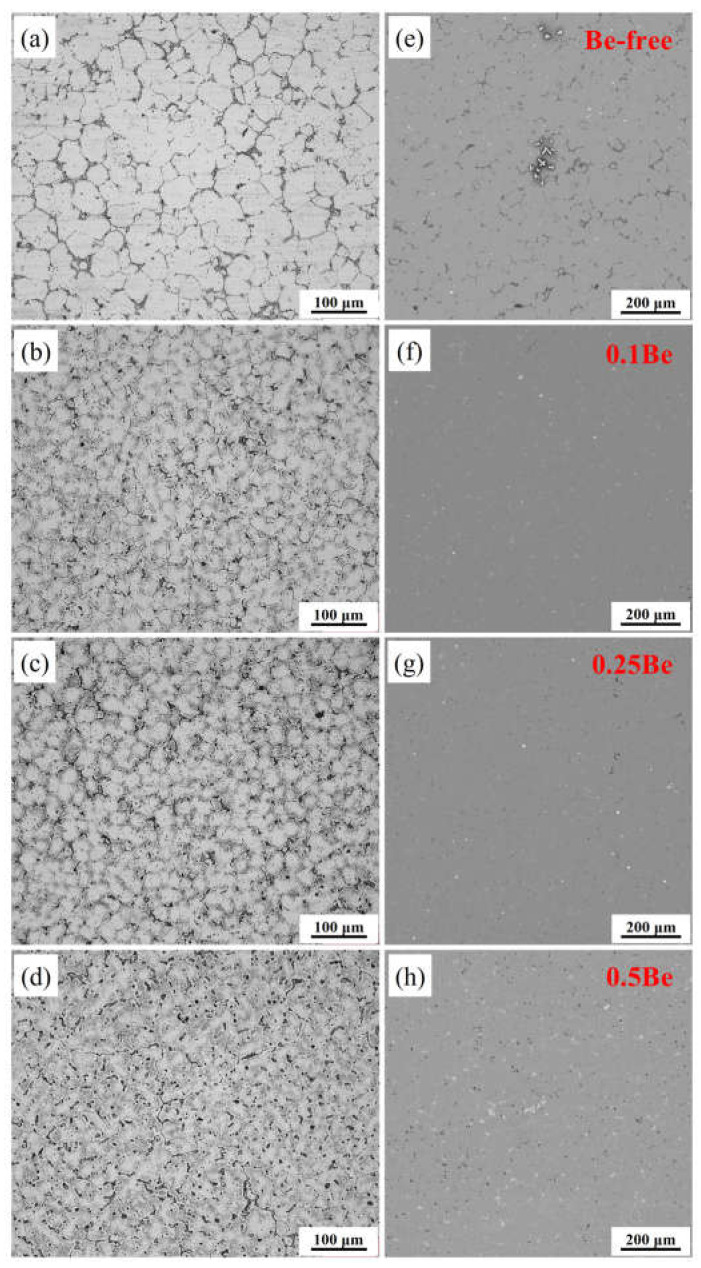
OM and SEM graphs of the as-cast ingots: (**a**,**e**) alloy 1; (**b**,**f**) alloy 2; (**c**,**g**) alloy 3; (**d**,**h**) alloy 4.

The OM and SEM images of the as-cast ingots with different Be additions are shown in Figure 5. The OM images present varying degrees of dendritic segregation within the grains of all of the alloys, where their as-cast grain morphology is quite different. Alloys 1–3 exhibit an equiaxed-grain structure, while alloy 4 shows a distinctive dendritic grain structure. The average grain sizes of alloys 1 and 2 are statistically counted to be 41.3 μm and 22.6 μm, while alloy 3 and alloy 4 are 29.4 μm and 28.7 μm, respectively. Excessive addition of Be (0.50 wt.%) causes the alloy grains to become particularly coarse and the grain morphology changes from equiaxed to dendritic.

According to the SEM images of the as-cast alloys with various Be content, Be-free alloy 1 has a more distinctive element segregation and a higher number density of white non-equilibrium crystalline phases. In contrast, the addition of trace Be (0.1 wt.%) significantly reduced the element segregation in Alloy 2, with only some small white insoluble secondary phases. It indicates that the addition of trace Be can help to alleviate the elemental segregation and reduce the grain size in the as-cast Al-Mg-Li alloy. The grain structure of Alloy 3 is similar to that of Alloy 2, but it has more black-dotted secondary phases. Alloy 4 shows more black-dotted secondary phases and white non-equilibrium crystalline phase areas. With the increase of Be content, the density of the black punctate solidification phase increases, while the white non-equilibrium crystalline phase changes from a discrete distribution to a continuous distribution.

#### 3.3.2. Distribution Form of Be Oxides

**Figure 6:**
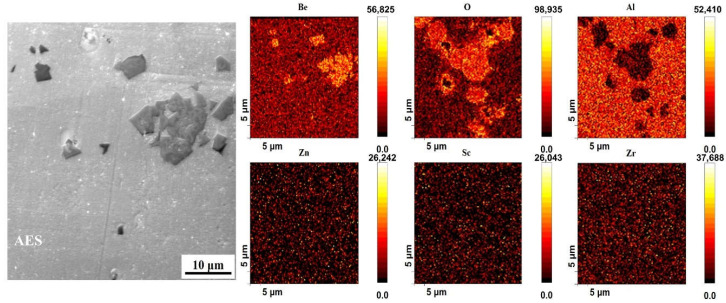
AES image and corresponding scan mapping analysis of as-cast alloy 4.

**Figure 7:**
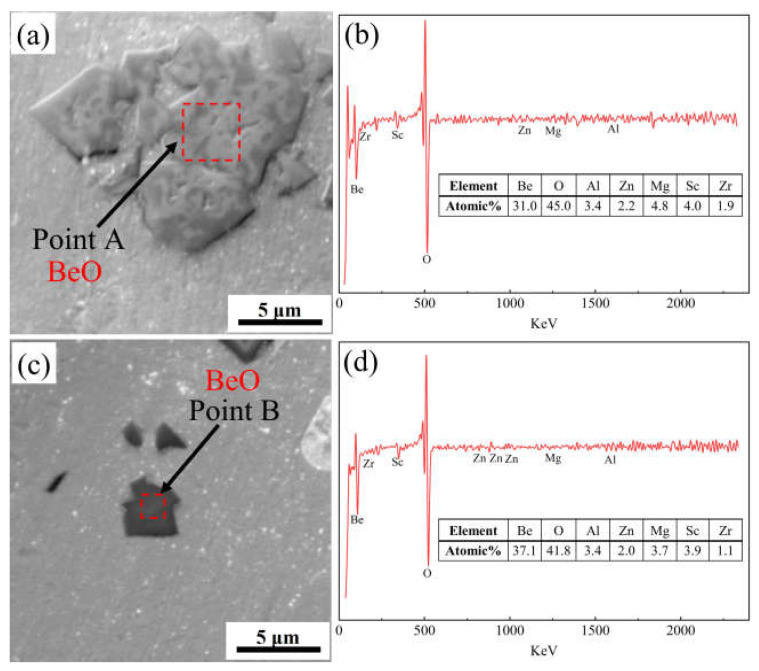
(**a**,**c**) High magnification AES image and (**b**,**d**) corresponding point scan analysis of the as-cast alloy 4.

To further uncover the distribution of Be addition in the alloy, the as-cast alloy 4 was analyzed using Auger electron spectroscopy (AES), and the results are shown in Figure 6 and Figure 7. According to the results of the AES scan mapping analysis of alloy 4 (Figure 6), Be is present in the as-cast alloy 4 in two main forms, a secondary phase with a large size and layered stacking distribution, and a secondary phase with a smaller size and dispersed distribution. The distribution areas of both phases are rich in O elements, so it can be tentatively concluded that both phases are oxides of Be. Subsequently, AES point scanning results of the two Be containing phases in alloy 4 revealed that the main constituent elements of both phases are Be and O (Figure 7). The atomic ratios of Be and O in the large-size Be containing phase are 31 at.% and 45 at.%, while in the small-size Be containing phase are 37 at.% and 41 at.%, which are recognized to be BeO. Therefore, the addition of 0.50 wt.% Be results in the formation of large amounts of Be oxides, which deteriorate the properties of the alloy.

#### 3.3.3. Recrystallization Behaviors

**Figure 8:**
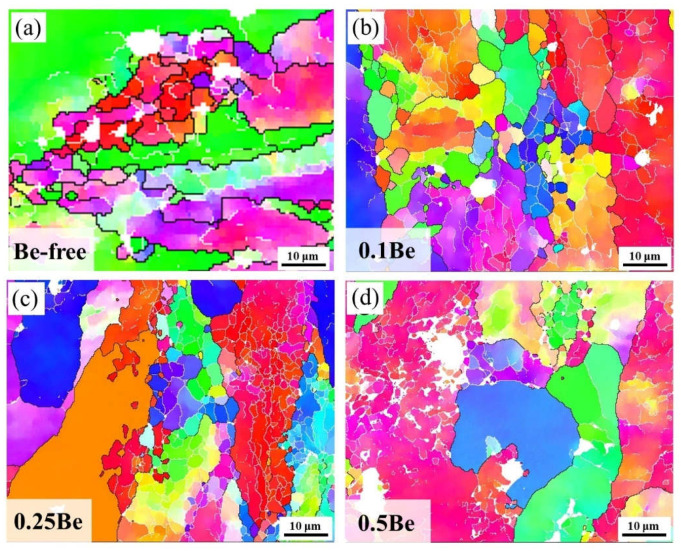
The IPFs map of hot-rolled sheets after solid solution treatment: (**a**) alloy 1; (**b**) alloy 2; (**c**) alloy 3; (**d**) alloy 4.

The inverse pole figures (IPFs) map of hot-rolled sheets after solid solution treatment of alloys with different Be additions after solid solution treatment are presented in Figure 8. The orientation deviation (θ) of low-angle grain boundaries, which is the white line in the IPFs map, is in the range of 2–15°. The θ of high-angle grain boundaries is the black line in the IPFs map and is in the range of θ > 15°. The grain structures of the alloys with different Be contents after solid solution treatment are quite different. It is obvious that alloy 1 retains distinctly elongated grains, and the alloys that add Be exhibit more low-angle grain boundaries than that of the Be-free alloy 1. Thus, the research of recrystallization behaviors is necessary for the above different grain structures.

**Figure 9:**
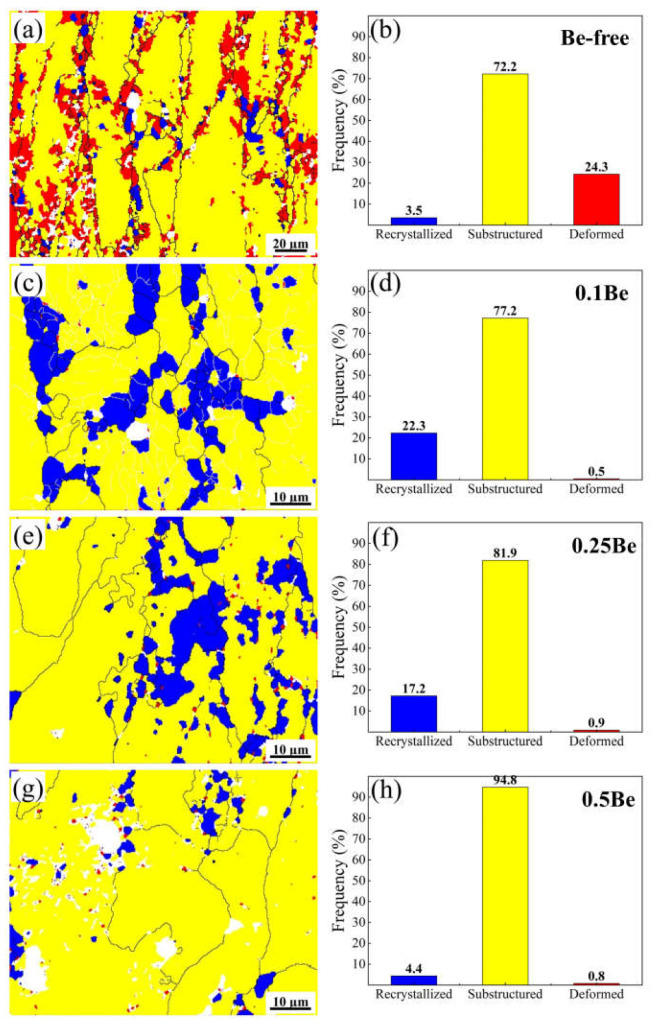
The recrystallization fraction of hot-rolled sheets after solid solution treatment: (**a**,**b**) alloy 1; (**c**,**d**) alloy 2; (**e**,**f**) alloy 3; (**g**,**h**) alloy 4.

The distribution statistics of the recrystallization fraction of hot-rolled sheets of alloys with various Be content after solid solution treatment are presented in Figure 9. After the solid solution, the grain morphology of the Be-free alloy 1 consists mainly of sub-structured and deformed grains, with only partially recrystallized grains during the dynamic recovery and dynamic recrystallization, and the proportions of the recrystallized grains, sub-structured grains, and deformed grains are 3.5%, 72.2%, and 24.3%, respectively. While the solid solution treated alloy 2, containing 0.1 wt.% Be, has more recrystallized grains and more substructures. Compared to alloy 1, the proportion of recrystallized grains increased, and the proportion of deformed grains reduced remarkably, with the proportion of recrystallized, sub-structured, and deformed grains being 22.3%, 77.2%, and 0.5%, respectively. When the Be content increases to 0.25 wt.%, the degree of recrystallization in Alloy 3 decreases, the sub-structure grains increase, the deformed grains remain at the level of alloy 2, and the proportions of recrystallized grains, sub-structured grains, and deformed grains are 17.2%, 81.9%, and 0.9%, respectively. As the Be content continues to elevate, the degree of recrystallization in alloy 4 decreases further, the proportion of substructures increases, and the deformed grains decreases, with the proportions of recrystallized, sub-structured, and deformed grains being 4.4%, 94.8%, and 0.8%, respectively.

In summary, the minor addition of 0.1 wt.% Be into the Al-Mg-Li alloy can significantly enhance its recrystallization degree, but with the continued increase of Be addition, the recrystallization degree decreases.

#### 3.3.4. Precipitation Behaviors

**Figure 10:**
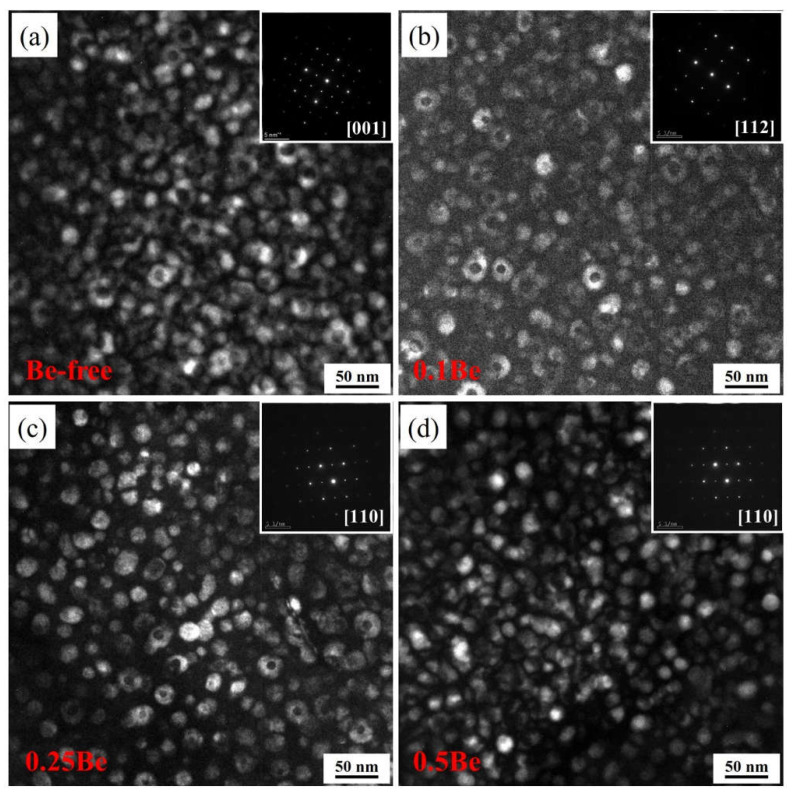
TEM dark field images of alloy in T6 aging condition: (**a**–**d**) alloys 1–4.

Figure 10 exhibits the dark-field images of alloys 1 to 4 after aging at 145 °C for 48 h. There is a small amount of uniformly distributed δ′ phase and some discretely distributed Al_3_(Sc, Zr)/Al_3_Li core-shell composite particles in the grains, but there are some differences in the morphology and number density of these phases of each alloy.

The δ′ phase in alloy 1 presents as incomplete regular particles, while with the addition of Be, the δ′ phase in alloys 2–4 shows a more uniform and regular spherical shape. A certain number of dispersive distributed Al_3_(Sc, Zr)/Al_3_Li core-shell structure composite particles can be seen in alloys 1 to 3, but only very few Al_3_(Sc, Zr)/Al_3_Li particles are seen in alloy 4, which own the highest Be content, interspersing with many δ′ phases. It can be assumed that the addition of Be would change the morphology of the δ′ phase in the Al-Mg-Li alloy, while the formation of Al_3_(Sc, Zr)/Al_3_Li core-shell structure composite particles is inhibited when the Be content increases to a certain degree, which results in a decrease in strength.

**Figure 11:**
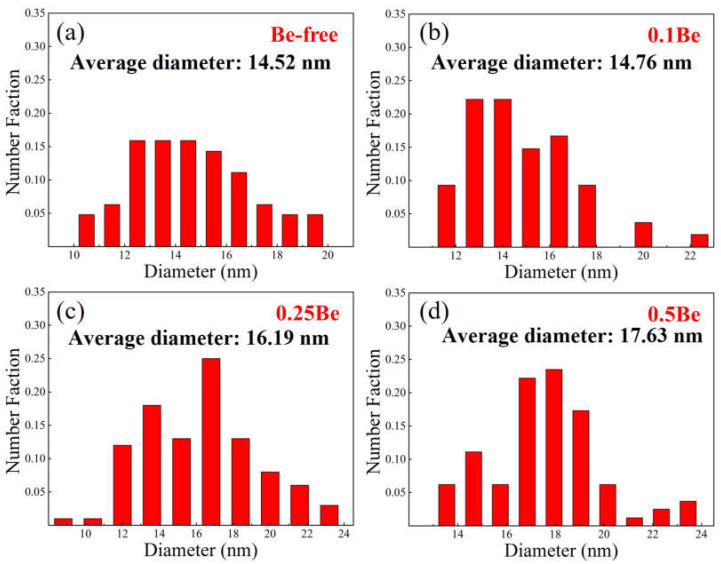
Size distribution of precipitate particles in the alloy: (**a**–**d**) alloys 1–4.

The TEM images of alloys 1–4 were then further analyzed and the size distribution of the δ′ precipitates in each alloy were calculated, and the results are shown in Figure 11. The statistical results show that the average diameters of δ′ phase in alloys 1 to 4 are 14.52 nm, 14.76 nm, 16.19 nm, and 17.63 nm, respectively. The average size of δ′ phase in alloy 1 and alloy 2 did not differ much, while the average size of the δ′ phase increased with the increase of Be addition. The addition of excessive Be in the Al-Mg-Li alloy would cause the coarsening of the δ′ phase and make the strength and plasticity decrease.

**Figure 12:**
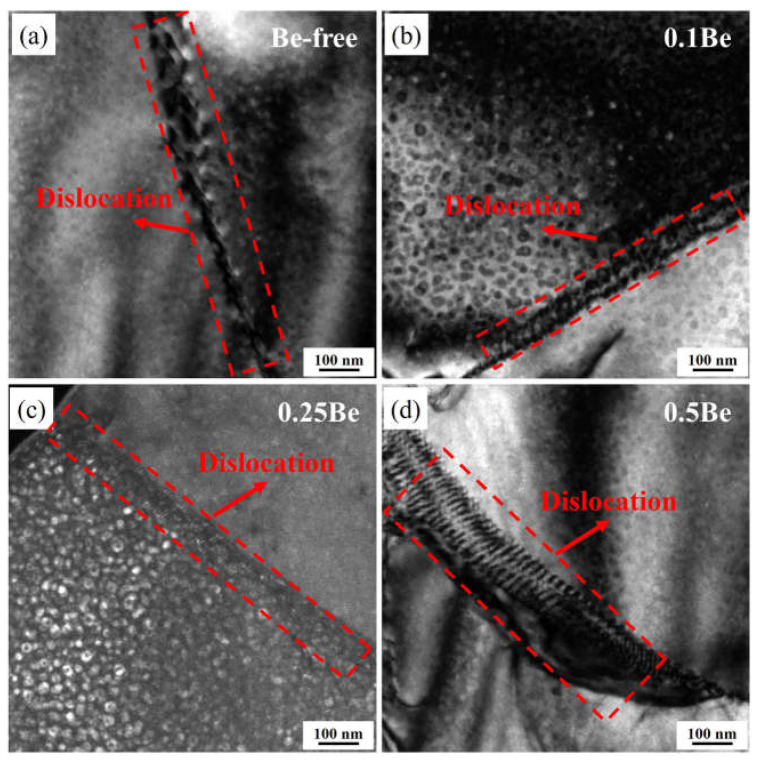
TEM images of dislocation pile-up in alloy after tensile test: (**a**–**d**) alloys 1–4.

It is found that dislocation pile-up existed at the grain boundary after the tensile test, and the TEM dark field images of alloy 1 to alloy 4 are shown in Figure 12. It is obvious that alloy 4 exhibits the most serious dislocation pile-up, followed by alloy 1, alloy 2, and alloy 3, which may be due to the small volume fraction of Al_3_(Sc, Zr)/Al_3_Li composite particles in alloy 4. Moreover, the δ′ phase has low strength contribution and is easily cut through by dislocations, indicating its weak ability to hinder the dislocation movement. The dislocations are severely accumulated at the grain boundaries. The dislocation pile-up will lead to a significant increase in the stress of the dislocation source, resulting in stress concentration. The dislocation pile-up to a certain level will produce cracks along the grain boundary, reducing the plasticity of the alloy.

**Figure 13:**
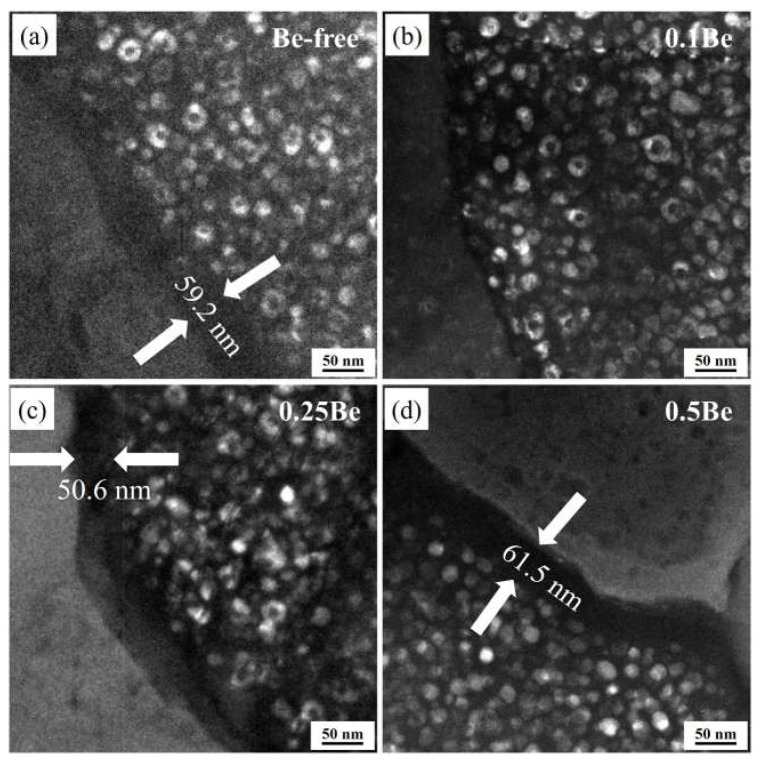
TEM images of grain boundaries of alloy in T6 aging condition: (**a**–**d**) alloys 1–4.

Figure 13 presents the dark-field images of the grain boundary of the alloys with different Be content after aging treatment at 145 °C/48 h. The morphology of the precipitation-free zones (PFZs) of the alloys differed notably, with Alloy 2 having no obvious PFZs, while Alloy 1, Alloy 3, and Alloy 4 exhibited wide PFZs, with their PFZ widths of 59.2 nm, 50.6 nm, and 61.5 nm, respectively. This indicates that the addition of 0.1 wt.% Be can significantly reduce the width of the PFZ and enhance the IGC resistance, whereas the PFZ of the alloy becomes wider as the Be content continues to increase, resulting in a weakening of the corrosion resistance.

## 4. Discussion

### 4.1. The Effect of Be Content on the Mechanical Properties

Be is similar to Li in atomic number and atomic weight, and its addition to Al-Mg-Li alloys not only reduces the density but also has a positive effect on the comprehensive properties, such as the mechanical properties and the anti-corrosion performance. The addition of trace Be element into Al-Mg-Li alloys significantly decreases the oxidative burnout of Mg and reduces inclusions during the casting process, due to the diffusion of Be into the alloy melt to form an oxide film.

The yield strength of polycrystalline materials can be seen as a combination of different strengthening effects, which can be calculated by Equation (1) [28,29,30,31],

(1) $$\Delta \sigma =M\Delta \tau +\sigma_{GB}$$

where $\sigma_{GB}$ stands for the strength factor influenced by grain boundary, and ∆*τ* stands for the critical shear stress. ∆τ can be presented as follows Equation (2):

(2) $$\Delta \tau =\tau_{0}+\tau_{ss}+\sqrt{\tau_{D}^{2}+\tau_{P}^{2}}$$

where $\tau_{0}$ stands for the internal stress of pure Al, and $\tau_{ss}$ stands for the strength contribution of the solid solution atoms. In general, the solid solution strengthening contribution is weak for heat-treated aluminum alloys. $\tau_{D}$ stands for the strengthening contribution of dislocations hardening, and $\tau_{P}$ stands for the strengthening contribution of precipitates.

In this work, the grain boundary strengthening mechanism and precipitation strengthening mechanism were considered. The trace amount of Be addition leads to a remarkable increase in recrystallization degree, which increases the value of $\sigma_{GB}$, and in the number density of Al_3_(Sc, Zr)/Al_3_Li core-shell structure composite precipitates. These composite particles cause the dislocation cutting mechanism from a shearing mechanism to an Orowan by-passing mechanism. The value of $\tau_{P}$ is elevated, thus it increases ∆*τ*, and therefore enables strength enhancement. There are remarkable differences in grain boundary strengthening effects of various Be-content alloys. The change of precipitates causes the cutting mechanism to switch and causes the precipitation strengthening effect to decrease, thus the grain boundary strengthening effect and the shift in precipitates play an important role in the increasing strength.

The experimental results presented that the addition of 0.1–0.25 wt.% Be can notably refine the grains of Al-Mg-Li alloy and elevate the strength, but the addition of 0.50 wt.% Be causes a significant growth in the grain size of Al-Mg-Li alloy and generates excessive Be oxides in the alloy. According to the experimental results in Figure 9 and Figure 10, the 0.50 wt.% addition of Be leads to the coarsening of the δ′ phase and inhibits the generation of Al_3_(Sc, Zr)/δ′ composite particles, which reduces the properties of the alloy. The addition of Be also elevates the thermal cracking resistance of the alloy, causing beryllium to be dissolved in some phases of the alloy, thus improving the thermal stability. The addition of 0.1 wt.% Be obviously promotes the recrystallization of Al-Mg-Li alloys, and then the recrystallization of the alloys decreases with an increase in the Be content, and the further addition of Be inhibits the recrystallization of Al-Mg-Li alloys.

### 4.2. The Effect of Be Content on the Corrosion Performance

As mentioned above, the Be addition affects the evolution of the microstructure and the performance of alloys. It is more relevant that the corrosion behavior of alloys is also influenced by precipitates inside the grains and at grain boundaries [32]. Previous studies have shown that the anti-corrosion performance of Al alloys is mainly related to grain boundary precipitates (GBPs), which in turn are affected by the structure of grain boundary [32,33]. Based on the study [34], the increase of low-angle grain boundaries can improve the corrosion resistance of Al alloys by inhibiting the precipitation of grain GBPs. Besides, the width of the PFZ within the alloy grains also has an indispensable effect on its corrosion resistance. The wider the PFZ, the worse the anti-corrosion performance. The reason for this is its electrochemical nature, which is the potential difference between the intermetallic phase and the Al matrix.

According to the experimental results in Figure 3 and Figure 4, it can be seen that the addition of 0.1 wt.% Be could remarkably enhance the corrosion resistance of Al-Mg-Li alloy. It exhibits more low-angle grain boundaries and simultaneously reduces the width of PFZ of the grain boundaries (Figure 8). Intergranular corrosion occurs along grain boundaries, the more low-angle grain boundary with low energy, the better the resistance to IGC. The alloy with 0.1 wt.% Be addition exhibits more uniform grains and no obvious elongated grains, leading to better resistance to exfoliation corrosion. In contrast, the Be-free alloy 1 and alloy 4 with 0.50 wt.% Be addition shows obvious elongated grains, thus owning a worse resistance to exfoliation corrosion. Moreover, the addition of 0.50 wt.% Be further deteriorates the IGC resistance of Al-Mg-Li alloys.

## 5. Conclusions

To further develop the Al-Mg-Li alloys, four Al-Mg-Li alloys with different Be additions (Be-free, 0.1, 0.25, 0.50 wt.%) were prepared in this work. The tensile properties, corrosion behavior, and microstructure of these alloys were systematically investigated. The main conclusions are as follows:

(1) The addition of 0.1–0.25 wt.% Be could remarkably refine the as-cast grain structure and alleviate the dendritic segregation. However, with the increase of Be content to 0.50 wt.%, the as-cast grain size increases, and the grain morphology changes from equiaxed grains to dendritic grains, while Be oxides of two forms are generated in the as-cast alloy, which reduces the final performance.

(2) The alloy with a trace addition of Be (0.1 wt.%) presents the optimal tensile property compared to the others. The addition of trace Be in the casting process leads to grain refinement while avoiding the generation of excessive Be oxides. Moreover, it promotes the recrystallization behavior during the solid solution process, while the optimal effect of the precipitation strengthening of the dispersive δ′ precipitates and Al_3_(Sc, Zr)/δ′ composite particles is obtained after the aging process.

(3) The trace addition of 0.1 wt.% Be notably increased the corrosion resistance of Al-Mg-Li alloy, but the excessive addition of Be will deteriorate the anti-corrosion performance. The addition of 0.1 wt.% Be can significantly reduce the PFZ width and enhance the IGC resistance, whereas the PFZ becomes wider as the Be content continues to increase, resulting in a weakening of the corrosion resistance.

## Figures and Tables

**Table 1:** Chemical composition of Al-Mg-Li alloys studied in this work (mass. %).

Alloy	Mg	Li	Be	Sc	Zr	Al
1	6.00	1.90	0	0.10	0.10	Bal
2	6.00	1.90	0.10	0.10	0.10	Bal
3	6.00	1.90	0.25	0.10	0.10	Bal
4	6.00	1.90	0.50	0.10	0.10	Bal

**Table 2:** Tensile properties of the alloys with different Be additions after T6 aging treatment.

Alloy	YS (MPa)	UTS (MPa)	El (%)
1	495	331	8.3
2	530	370	9.2
3	502	338	9.7
4	464	309	6.5

## Data Availability

The raw/processed data required to reproduce these findings cannot be shared at this time as the data also forms part of an ongoing study.
